# Self-Awareness of Goals Task (SAGT) and Planning Skills: The Neuroscience of Decision Making

**DOI:** 10.3390/brainsci13081163

**Published:** 2023-08-03

**Authors:** Michela Balconi, Laura Angioletti, Carlotta Acconito

**Affiliations:** 1 International Research Center for Cognitive Applied Neuroscience (IrcCAN), Catholic University of the Sacred Heart, Largo Gemelli 1, 20123 Milan, Italy; michela.balconi@unicatt.it (M.B.); carlotta.acconito1@unicatt.it (C.A.); 2 Research Unit in Affective and Social Neuroscience, Department of Psychology, Catholic University of the Sacred Heart, Largo Gemelli 1, 20123 Milan, Italy

**Keywords:** decision making, self-awareness, goals, behavioral neuroscience, EEG

## Abstract

A goal’s self-awareness and the planning to achieve it drive decision makers. Through a neuroscientific approach, this study explores the self-awareness of goals by analyzing the explicit and implicit processes linked to the ability to self-represent goals and sort them via an implicit dominant key. Thirty-five professionals performed a novel and ecological decision-making task, the Self-Awareness of Goals Task (SAGT), aimed at exploring the (i) self-representation of the decision-making goals of a typical working day; (ii) self-representation of how these goals were performed in order of priority; (iii) temporal sequence; and (iv) in terms of their efficacy. Electrophysiological (i.e., alpha, beta, and gamma band), autonomic, behavioral, and self-report data (General Decision Making Style and Big Five Inventory) are collected. Higher self-awareness of goals by time as well as efficacy and the greater activation of alpha, beta, and gamma bands in the temporoparietal brain area were found. Correlations reported positive associations between the self-awareness of goals via a time and dependent decision-making style and a conscientious personality, but also between the self-awareness of goals via an efficacy and rational decision-making style. The results obtained in this study suggest that the SAGT could activate recursive thinking in the examinee and grasp individual differences in self-representation and aware identification of decision-making goals.

## 1. Introduction

Each individual makes decisions in every moment of his daily life, both in his private life and in professional contexts. Specifically, in organizational settings, professionals and managers are called to make decisions dynamically and quickly, taking into consideration multiple sources of information to formulate flexible action plans and balance their multiple work goals.

To choose an optimal course of action, select and analyze a large amount of information and multiple alternatives, make strategic adjustments to changes in a situation, and correctly define the objectives to be achieved, it is crucial for decision makers to develop continuous awareness about themselves and the context around them [1,2,3,4].

An individual’s knowledge about one or more aspects of the self [4], as well as the ability to identify, process, and store information about oneself [5], falls under the definition of the concept of “self-awareness”. Regarding the decision-making process, early research proposed that states of elevated self-awareness led people to be more aware of their own goals [6,7], in addition to their thoughts, feelings, and behavior.

Given the importance of these capabilities, various research has been conducted in different disciplinary fields, especially in organizational psychology [8]. Self-awareness of one’s goals, together with the capacity to predict the possible obstacles to achieving them, indeed drive a decision maker in his/her decisions [2]. Each step of the decision-making process, such as (i) problem identification and goal definition, (ii) information gathering, (iii) elaboration and prediction, (iv) strategic and tactical planning, (v) decision making and action, and (vi) the evaluation of outcomes with a possible modification of strategy, can be marked by different levels of self-awareness.

Focusing on the first step, problem identification and goal definition have long been considered some of the most critical steps in making good decisions [9], since these first steps influence subsequent performance. Objectives, in fact, drive attention and effort toward activities relevant to the achievement and satisfaction of the goal itself in addition to affecting the amount of cognitive, physiological, and subjective effort put into the activity. Goals also influence effort in relation to the ability to control the time spent on each task, but also indirectly by leading to the arousal, discovery, and/or use of task-relevant knowledge and strategies [3].

Within this initial stage of the decision-making process, self-awareness plays a primary role by leading individuals to identify what the goals to be achieved are in relation to different tasks. In addition, the ability to have a clear and conscious vision of one’s goals is also responsible for self-regulatory processes that support the achievement of the goals themselves [10]. Specifically, individuals in a more self-aware state more correctly identify goals and monitor their pursuit more actively, identifying and reducing any discrepancies between them [7].

Indeed, as a result it is essential for an individual to be proficient at self-representing his/her own goals (by being able to list them, for instance), as well as sorting them according to different criteria [11].

The level of ability to self-represent one’s goals could provide insights into the most relevant factors within the decision-making process and highlight the implicit dominant key guiding the self-awareness of daily tasks and objectives—i.e., whether an individual is mainly guided by decision priority, timing, or efficacy when recalling his/her own objectives. The target environment must also be taken into consideration when self-representing decision-making goals. For instance, making a decision in a complex context, such as a professional context, could be a situation featuring stressful and unplanned situations, unexpected changes, and constrained time.

With reference to the first criteria, goal prioritization, it is a key concept for understanding the pursuit of different goals and can be defined as the temporary increase in the importance attributed to one or more goals and the resources directed toward them to facilitate their execution [12]. Specifically, priority could be assigned to goals that have a high informational value, high affective value, and high expectancy [12]. Having a good awareness and adequate representation of one’s prioritization of goals has previously been associated with a better translation of goals into actions [13], but also with a greater likelihood of achieving results, especially when deadlines are involved [14]. Prioritization leads to a higher commitment of time and energy to pursuing established goals [15], inhibiting those that are not prioritized [12].

Secondly, the ability to prioritize one goal over another in accordance with the time variable is linked to goal shielding, a phenomenon that, in accordance with Orehek and Vazeou-Nieuwenhuis’ theory [16], is a self-regulation strategy that facilitates the management of multiple goals. The deadline of a goal, in fact, is another important factor in deciding how to prioritize an entire set of goals [17]. The importance of prioritization according to time also appears to be more important than the distance of the goal. In accordance with the research of Schmidt and DeShon (2007), in fact, people normally tend to prioritize the goal furthest from completion, but as the deadline for a given goal approaches the latter is prioritized more [18].

Thirdly, the ability to order one’s goals by efficacy involves the need to develop an awareness of one’s effectiveness, identifying goals achieved and those yet to be achieved. With respect to the efficiency factor, previous studies have shown that the perceived difficulty attributed to a goal is, in fact, one of the possible drivers of goal prioritization. Specifically, if a goal is perceived as too difficult to reach, it is postponed to the advantage of a more achievable goal [8,19].

Overall, the ability to represent one’s own goals more or less effectively could also affect an individual’s decision-making style. Indeed, by investigating an individual’s level of self-awareness of their own goals, it is possible to understand how often a person adopts a more reflective or impulsive approach to decision making. Specifically, having a good self-representation of one’s goals can be understood as an indication of a mostly rational decision-making style, in that a person requires the careful consideration of all alternatives and the various related efficacies, but also a conscious prioritization of activities, to have a good awareness of his/her goals.

From a methodological perspective, some studies have previously tried to define how people make decisions via the use of questionnaires that allow the defining of a person’s decision-making style, such as the General Decision Making Style [20], the Big Five Inventory [21], or the Maximization Scale [22]. However, with this approach it is possible to focus exclusively on the explicit and relatively conscious parts of decision-making styles, or what people believe and refer to as their own knowledge, which may be biased by a variety of factors, such as social desirability.

A significantly different approach, which is that of neuroscience, consists of asking individuals to perform a task related to their ability to self-represent their decision-making goals and in the meantime collect the behavioral responses and neuroscientific correlates of this process. Indeed, the neuroscientific perspective, which enables researchers to focus not only on the explicit but also on the implicit components of a decision-making process, has recently started to contribute to understanding decision-making styles in all their complexity, specifically in the marketing field [23,24]. Indeed, neuroscientific tools, such as electroencephalograms [25] and autonomic measure recordings [26], allow the measurement of the typical neuro- and psycho-physiological responses of a person during the decision-making process, highlighting levels of cognitive effort and emotional engagement [27]. Electroencephalograms, in particular, provide information on the electrophysiological activity of the brain, highlighting the functional meaning of frequency bands to assess the possible load and cognitive effort required by decision-making processes. Specifically, greater activation in low-frequency bands (theta and delta) could be associated with emotional processes [28], while alpha and beta bands are indices of cognitive effort, active attention, and engagement [29,30]. On the other hand, autonomic measures, such as electrodermal activity (i.e., skin conductance level and response) and cardiovascular indices (i.e., heart rate), provide insight into emotional processes such as emotional engagement, arousal, and stress levels [31,32,33].

In the professional field, decision making has been explored through a neuroscientific perspective in different applied contexts [28,31,34,35,36]. Additionally, basic research has used EEG to investigate specific effort allocation during task prioritization. Although these studies show that the prioritization task requires cognitive control, evidenced by low-frequency band and alpha as well as beta band brain activity [37,38], no previous works have been conducted to explore the self-representation of decision-making goals as defined in our study. Furthermore, to the best of our knowledge, no previous neuroscientific studies have focused on the neurophysiological correlates of self-representing our own decision goals in a professional context and sorting them for priority, timing, or potential efficacy.

In order to compensate for the gap in the literature, this study aims to explore, with a neuroscientific approach, how individuals, particularly professionals, are characterized by different cognitive and emotional reactions connected to the awareness of one’s decision-making goals.

For the purpose of measuring the ability of a sample of professionals to list decision-making objectives, to have them evident and contextualized with respect to the present moment, we designed a novel experimental task to detect the self-awareness of one’s decision-making goals in individuals, named the self-awareness of goals task (SAGT). This task is composed of four different steps: (i) the self-awareness of the decision-making goals of the working day before (e.g., goals listing), (ii) the self-awareness of how these were performed in order of priority (i.e., sorting the goals in order of importance), (iii) the self-awareness of how these were carried out in a temporal sequence (i.e., sorting the goals into the order in which they were executed), and (iv) the self-awareness of these in terms of efficacy (i.e., sorting the goals according to what degree the person was able to accomplish them). To measure the self-awareness of one’s goals in a complex context, such as the professional one, a reduced time window was given to provide a behavioral response for each step.

Throughout the duration of the task, electrophysiological (EEG) and autonomic activity were detected to complement and enrich observations based on participants’ behaviors and responses. For each step, specifically, besides the number of decision-making goals indicated by the professional, response times (RTs) were also collected as an indirect measure of workload to assess the effort employed by participants for each self-representation step. Indeed, RTs allowed for the evaluation of participants’ responses considering the cognitive cost of the identification and decision-making processes that underlie the self-representation of the decision-making goals. In particular, longer RTs reflect greater task-related effort and, in turn, could suggest greater effort in the self-representation of goals.

In addition to the behavioral measures and to have a comprehensive overview of the self-awareness of the decision-making goals, the self-report measures of the General Decision Making Style and 10-item Big Five Inventory were administrated to measure a participant’s decision-making style and personality traits. With these self-report instruments, it is possible to compare the level of decision-making self-representation, investigated mainly via implicit measures, with the decision-making style detected via validated questionnaires referring to the most conscious and aware part of one’s decision-making processes.

In terms of specifics, it was expected that the level of self-representation of decision-making goals in a group of professionals called upon to operate and make decisions in a complex work environment may be characterized by a style more focused on ordering their goals by time or by efficacy. This style will be characterized by a higher behavioral score in the ability to self-represent how goals were achieved in temporal sequence and in terms of efficacy. At the same time, as an index of less cognitive effort, lower RTs are expected in these two specific steps of the SAGT compared to the other steps.

Regarding EEG results, it was hypothesized that a general decrease in the alpha band and an increase in the beta band in the frontal areas were indicators of cognitive effort and engagement during this novel experimental task [29,30]. Concerning autonomic measures, it was estimated that high levels of skin conductance level and heart rate would be found, especially in the different sorting tasks. High levels of these autonomic indices, in agreement with the literature, can be interpreted as cues of stress and emotional engagement [33,39], which may result from both the difficulty of having a clear representation of the different variables of priority, efficacy, and time attributable by the different goals as well as from awareness with respect to the need to complete the SAGT in the shortest RT.

Finally, it was expected that high values in the self-representation of decision-making goals in terms of timing would correlate with a conscientious (10-item Big Five Inventory score) decision-making style, since completing different tasks on time requires good organizational skills and planned behavior. Similarly, high values in the self-representation of decision goals in terms of efficacy would correlate with a more rational decision-making style (General Decision Making Style score), typical of a person who is aware that a given choice may lead to outcomes other than the ones predetermined.

In conclusion, this research aimed to explore professionals’ cognitive and affective responses connected to the self-awareness of one’s decision-making goals by exploiting, for the first time, a combined behavioral and neurophysiological approach. The decision to conduct this study on a sample of professionals was motivated by the fact that they are involved in complex decision-making processes on a daily basis and strive to achieve as well as maintain optimal performance under changing conditions, emphasizing the need to adapt decisions to real-world situations and the constraints imposed by continuous change and ambiguity.

## 2. Materials and Methods

### 2.1. Sample

The sample consisted of 35 professionals (female = 22; male = 13), with ages ranging from 24 to 59 years old (mean age = 38.29, standard deviation of age = 9.53), working in the managerial departments of a large service company in Italy. All of the participants had been employed in the same job position for about two years at the time of the experiment. This precaution was introduced to avoid including participants who were more stressed due to, for instance, job changes or an increase in workload while shifting to new tasks or duties. Participants belonged to distinct internal departments with different specializations (e.g., human resource management, training and professional learning, engineering and maintenance management, service quality monitoring, infrastructure management, and others), so as to not only focus on one single professional specialty.

The sample was also defined according to the following exclusion criteria: (i) severe levels of depression, (ii) history of psychiatric or neurology disorders, (iii) abnormal short- and long-term memory, (iv) low global cognitive functioning, and (v) undergoing a concurrent therapy based on psychoactive drugs that could alter cognitive or decisional skills. Finally, all participants had normal-to-corrected vision.

All participants signed written informed consent and participated voluntarily without receiving any compensation. The research protocol has been approved by the Ethics Committee of the Department of Psychology, Catholic University of the Sacred Heart, Milan, Italy, and conducted in accordance with the Helsinki Declaration (2013).

### 2.2. Procedure

The experiment took place in a quiet dedicated room at the participants’ workplace, to preserve the everyday working context of the participants and, in the meantime, improve the ecological validity of the data collection.

The participants were asked to sit on a comfortable chair in front of a PC monitor placed about 80 cm away from them. After signing the written informed consent, a non-invasive EEG and autonomic measures recording device for collecting EEG and autonomic responses at rest and during task execution were applied to a participant. A 120 s eyes open baseline was collected before participants were given the instruction to perform the experimental task. At the end of the task, the General Decision Making Style and 10-item Big Five Inventory were administrated to collect self-report data. The experimental procedure had a duration of about 10 min.

#### 2.2.1. Behavioral Measures: Experimental Task and Data Processing

The participants were required to execute a novel ecological decision-making task, the SAGT, designed to evaluate the ability and modality of the self-representation of one’s decision-making goals and administered via a web-based survey and experiment management platform (Qualtrics XM platform; Qualtrics LLC, Provo, UT, USA).

Specifically, the SAGT is composed of four different steps:

(i) The self-representation of the decision-making goals of the working day before (i.e., goal listing).
(ii) The self-representation of how these were performed in order of priority (i.e., sorting the goals in order of importance).
(iii) The self-representation of how these were carried out in temporal sequence (i.e., sorting the goals in the order in which they were executed during a day).
(iv) The self-representation of these in terms of efficacy (i.e., sorting the goals according to how much the person was able to accomplish them).

**Figure 1:**
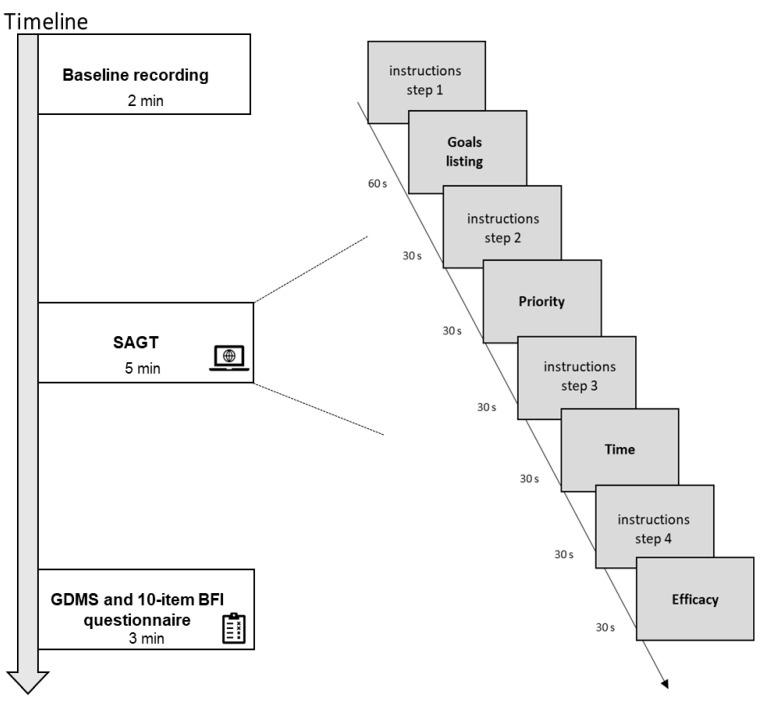
Experimental procedure and the SAGT. The figure shows the experimental procedure and the SAGT steps. Throughout the duration of the task, EEG and autonomic activity were detected to complement and enrich observations based on participants’ behavioral data. SAGT: Self-Awareness of Goals Task; GDMS: General Decision -Making Style; BFI: Big Five Inventory.

In the first step (i.e., “goal listing” in Figure 1), after an instruction screen, participants were asked to refer to the last working day and to list in a text box, as quickly as possible, all of the decision goals they had set for that particular day that involved a decision. To list the goals there was a time window of 60 s.

After pressing the “forward” button, participants were shown a screen asking them to recall previously written decision objectives and reorder them by priority, assigning the most important to the first position and the least important to the last position (i.e., “priority” in Figure 1). Then, in the next step, reordering by temporal sequence was required, where the first position is represented by the goal carried out first in the day and the last position by the one carried out last (i.e., “time” in Figure 1). Finally, in the last phase of the task, participants were required to reorder the goals by efficacy: the goal with the best efficacy is represented in the first position, while the one with the worst efficacy is shown in the last position (i.e., “efficacy” in Figure 1). For each reordering phase (by priority, temporal sequence, and efficacy), participants were given a maximum time of 30 s.

The SAGT is administered under time pressure in order to make the decisional process cognitively demanding and to make RTs an informative and discriminative measure of the cognitive load and efficiency of information processing.

Throughout the duration of the task, electrophysiological and autonomic activity were detected to complement and enrich observations based on participants’ behaviors and RTs. For a description of the procedure and of the SAGT, see Figure 1.

With regard to behavioral data, for the first step of the task, the number of the listed goals and the RTs employed to list the goals were considered.

Additionally, for each step of the SAGT, the number of the sorted goals for the specific criteria (priority, time, and efficacy) and the RTs used to sort the goals were collected. As noted above, besides the actual responses and related scores, RTs were also collected as an indirect measure of workload to assess the effort imposed on participants by each decisional step.

Response scores and response times were, then, transcribed offline into a common metric scale ranging between 1 and 10, and used to compute the self-representation of the decision-making goals (Self-Repi), the self-representation of how these were performed in order of priority (Priority-Repi), the self-representation of how these were carried out in temporal sequence (Temporal-Repi), and the self-representation of these in terms of efficacy (Efficacy-Repi) indices. These indices, therefore, express measures related to the SAGT and provide insights with respect to each participant’s level of ability to self-represent his or her goals and the dominant implicit key that drives self-awareness of daily tasks and goals. Such indices were calculated through a mathematical algorithm based on the ratio between response scores and response times, as follows:

Self-Repi = N goals_dec_/RT_dec_

Priority-Repi = ∆priority_dec_/RT_dec_

Temporal-Repi = ∆time_dec_/RT_dec_

Efficacy-Repi = ∆efficacy_dec_/RT_dec_

where N goals_dec_ refers to the total number of goals listed by the participants converted into the decile metric scale, ∆priority_dec_ refers to the difference between the total goals listed in Step 1 and the total goals sorted by priority, ∆time_dec_ refers to the difference between the total goals listed in Step 1 and the total goals sorted by time, ∆efficacy_dec_ refers to the difference between the total goals listed in Step 1 and the total goals sorted by efficacy, and RT_dec_ refers to participants’ response times converted into the decile metric scale.

Considering these calculations, high scores and low RTs would lead to the highest Self-Repi, Priority-Repi, Temporal-Repi, and Efficacy-Repi values, mirroring, respectively, greater self-awareness of the decision-making goals of the working day before and self-awareness of how these goals were performed in order of priority, in temporal sequence, and of efficacy.

The basis for this computation is the assumption that shorter RTs would mark lower task-related effort due to either more effective appraisal and decision-making skills in goal self-representation or implicit preference for one of the three sorting criteria (i.e., priority, temporal sequence, or efficacy) when planning one’s goals.

#### 2.2.2. EEG Data Acquisition

A wearable electroencephalogram system with dry sensors (Muse^TM^ headband, version 2; InteraXon Inc., Toronto, ON, Canada) was employed to record the resting-state and task-related variations in EEG spectral activity (standard-frequency band power: delta, theta, alpha, beta, and gamma) in a non-invasive manner. Three electrodes were used as a reference, and the remaining four were placed in the frontal (AF7 and AF8, left and right forehead, respectively) and temporoparietal (TP9 and TP10, left and right ear, respectively) regions. The three reference electrodes, which were not used to capture brain signals, were located between the two input electrodes on the forehead and corresponded to the electrode position Fpz. The frontal and temporoparietal electrodes’ positioning in the Muse headband followed the international 10–20 system [40] and were made up of conductive material (silver) and silicon rubber, respectively. The system was equipped with an accelerometer, gyroscope, and pulse oximetry. Data were collected and transmitted via Bluetooth to a connected smartphone, using the mobile application Mind Monitor. Data were sampled at a constant of 256 Hz, and a 50 Hz notch frequency filter was applied. Mind Monitor automatically processes raw data by applying a fast Fourier transform (Hamming window: length 10%, 0.5 Hz) to obtain brain waves at different frequency bands, using the logarithm of the power spectral density of the raw EEG data coming from each channel. Participants were instructed to minimize eye blinks and movements. The following frequency bands were extracted from each channel of the recorded electrophysiological signals: delta (1–4 Hz), theta (4–8 Hz), alpha (7.5–13 Hz), beta (13–30 Hz), and gamma (30–44 Hz). All of the EEG power spectral density values collected by Mind Monitor were typically in the range of −1 to +1. Finally, the normalization-applied procedure consisted of the baseline (as the 120 s resting baseline was recorded at the start of the experiment) correction of the signal.

The decision to use the Muse^TM^ headband as a brain activity detection tool was based on the fact that it is a portable device that is easy to use in business contexts, thereby preserving the ecological validity of the study. Additionally, even though this device only consists of two electrodes placed in the frontal region (AF7 and AF8) and two in the temporoparietal region (TP9 and TP10), it still permits the investigation of key regions of interest, such as the bilateral frontal regions, widely involved in decision-making processes [41], and the bilateral temporoparietal brain regions, which are involved in self-representation mechanisms as well as in stimulus–context integration processes [42,43,44].

#### 2.2.3. Autonomic Data Acquisition

A Biopac MP 150 system (Biopac Systems Inc., Goleta, CA, USA) was used to collect resting-state and task-related variations in the autonomic activity.

Electrocardiogram activity was recorded continuously in lead one from two electrodes attached to the lower wrist, with the positive pole on the left arm and the negative pole on the right one. The electrocardiogram signal was sampled at 1000 Hz with Biopac Acknowledge 3.7.1 software (Biopac Systems Inc.). The electrocardiogram was converted into heart rate. The signal was low-pass filtered at 35 Hz and high-pass filtered at 0.05 Hz for motor and ocular artifacts.

Electrodermal activity was recorded via the electrodes for the skin conductance response and skin conductance level attached to the distal phalanges of the first and fifth fingers of the left hand. The signal was sampled at 1000 Hz and low-pass filtered at 10 Hz for motor, ocular, and biological artifacts.

Electromyographic activity was recorded from 2 electrodes attached to the corrugator muscle with 2 electrodes attached, with the positive pole above the left eyebrow and the negative pole on the center of the forehead. The signal was sampled at 500 Hz and low-pass filtered at 10 Hz.

#### 2.2.4. Self-Report Data Acquisition

The General Decision-Making Style [20] and 10-item Big Five Inventory scales [21] were adopted to collect self-report data on individuals’ decision-making styles and personality traits.

Specifically, the General Decision-Making Style defines an individual’s decision-making style according to five different styles: rational, intuitive, dependent, avoidant, and spontaneous. The rational style, in particular, is characterized by a comprehensive and exhaustive search for information, considering all alternatives and their consequences; the intuitive style is defined by a focus on global aspects and a tendency to decide on hunches; the dependent style identifies a person who prefers to receive suggestions and advice; the avoidant style defines the person who tends to avoid making decisions; and the spontaneous style is typical of those who prefer to make a decision as quickly as possible.

On the other hand, the 10-item Big Five Inventory is a ten-item scale designed to assess the Big Five personality dimensions in a very short amount of time. This tool specifically provides guidance about five different aspects of personality: extroversion (being enthusiastic and extroverted), agreeableness (being likeable and warm), conscientiousness (being organized and self-disciplined), emotional stability (being calm, stable, and balanced), and openness (being imaginative and open to new experiences).

### 2.3. Statistical Analyses

A set of repeated measures analyses of variance (ANOVAs) were applied to behavioral, EEG, and autonomic data, considering the entire sample.

A first ANOVA with Condition (4: goals listing, priority, time, and efficacy) as the within-subject factor was applied to the behavioral indices (Self-Repi, Priority-Repi, Temporal-Repi, and Efficacy-Repi).

Secondly, for EEG data, five ANOVAs with Condition (4: goals listing, priority, time, and efficacy), Localization (2: frontal and temporoparietal), and Lateralization (2: right; left) as the independent within-subject factors were performed for each frequency band (delta, theta, alpha, beta, and gamma).

Finally, six ANOVAs with Condition (4: goals listing, priority, time, and efficacy) as the within-subject factor was applied to each autonomic index (i.e., skin conductance level, skin conductance response, heart rate, and electromyography).

Pairwise comparisons were applied to the data in cases of significant effects. Simple effects for significant interactions were further checked via pairwise comparisons, and the Bonferroni correction was used to reduce potential biases of multiple comparisons. For all of the ANOVA tests, the degrees of freedom were corrected using the Greenhouse–Geisser epsilon where appropriate. Furthermore, the normality of the data distribution was preliminarily assessed by checking kurtosis and asymmetry indices. The size of statistically significant effects has been estimated by computing partial eta-squared (η^2^) indices.

Pearson correlations, with Bonferroni corrections for multiple comparisons, were applied to the behavioral indices (Self-Repi, Priority-Repi, Temporal-Repi, and Efficacy-Repi) and General Decision-Making Style as well as 10-item Big Five Inventory scores of the entire sample.

## 3. Results

### 3.1. Behavioural Results

**Figure 2:**
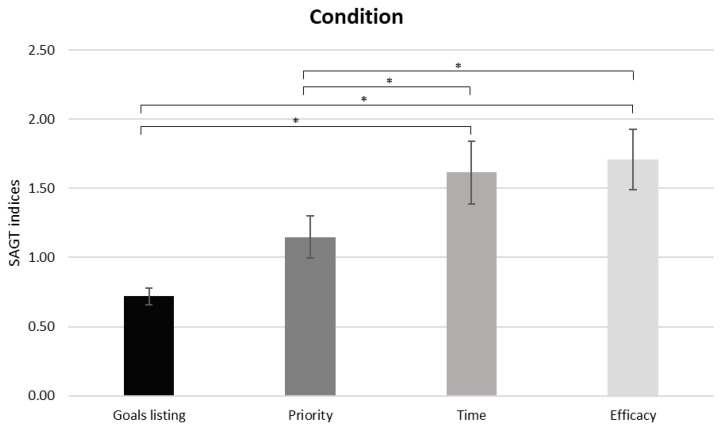
Behavioral results. The bar graph shows statistically significant differences in the behavioral SAGT indices for each SAGT step. Bars represent ± 1 standard error and stars (*) mark statistically significant comparisons.

As shown by the ANOVA for behavioral data, a significant main effect in the within-subject factor SAGT step was found (F[1,34] = 11,990, *p* ≤ 0.05, η^2^ = 0.261), for which an increase in Efficacy-Repi (behavioral index referring to the efficacy) compared to Self-Repi (*p* = 0.002) and Priority-Repi (*p* = 0.001) (the behavioral indices referring to goal listing and priority, respectively) was observed. Additionally, an increase in Temporal-Repi (the behavioral index referring to time) was found compared to Self-Repi (*p* = 0.005) and Priority-Repi (*p* = 0.008) (Figure 2).

### 3.2. EEG and Autonomic Results

**Figure 3:**
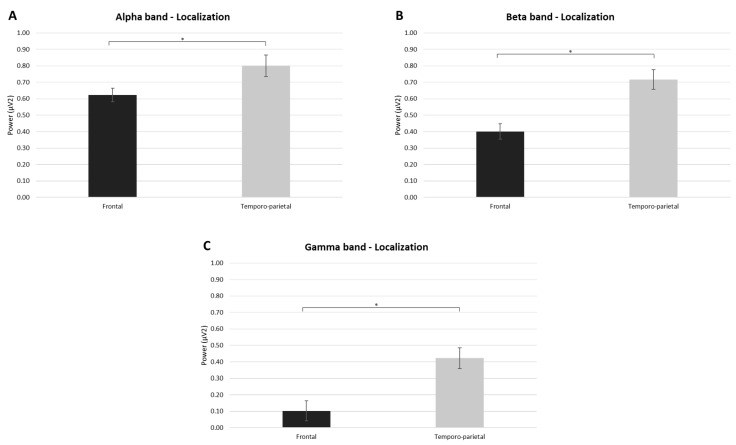
EEG results. (**A**) The bar graph shows significant differences for the alpha band in Localization. (**B**) The bar graph shows significant differences for the beta band in Localization. (**C**) The bar graph shows significant differences for the gamma band in Localization. For all graphs, bars represent ± 1 standard error and stars (*) mark statistically significant comparisons.

Regarding the ANOVAs performed on the EEG data, for the alpha band a significant main effect was found in the within-subject factor Localization (F[1,34] = 6942, *p* = 0.025, η^2^ = 0.410), with an increase in the activity of this band in the temporoparietal compared to the frontal area (Figure 3A).

Similarly, for the beta band, a significant main effect was found in the within-subject factor Localization (F[1,34] = 21,751, *p* ≤ 0.05, η^2^ = 0.685), with increased beta power in the temporoparietal compared to the frontal areas (Figure 3B).

Additionally, for the gamma band, a significant main effect was found in the within-subject factor Localization (F[1,34] = 25,721, *p* ≤ 0.05, η^2^ = 0.720), with an increase in the power of this band in the temporoparietal compared to the frontal locations (Figure 3C). No other significant differences were found, and no significant differences were found for the other EEG frequency bands (delta and theta bands).

Additionally, the ANOVAs performed on the autonomic indices (skin conductance level, skin conductance response, heart rate, and electromyography) did not show any significant difference.

### 3.3. Correlational Results

**Figure 4:**
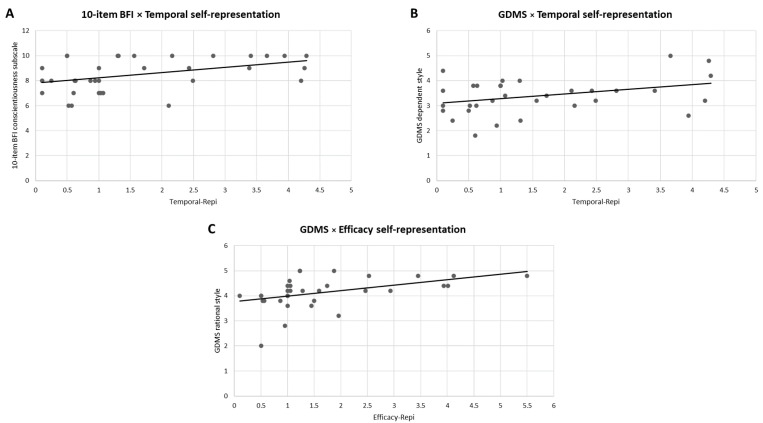
Pearson correlations. (**A**) The scatter plots display a significant positive correlation between the temporal representation SAGT index and conscientiousness. (**B**) The scatter plots display a significant positive correlation between the temporal representation SAGT index and a dependent decision-making style. (**C**) The scatter plots display a significant positive correlation between the efficacy representation SAGT index and a rational decision-making style.

The Pearson correlation performed between the SAGT behavioral indices (Self-Repi, Priority-Repi, Temporal-Repi, and Efficacy-Repi) and 10-item Big Five Inventory scores showed a positive correlation between Temporal-Repi and conscientiousness (r = 0.433, *p* = 0.009) (see Figure 4A).

The Pearson correlation performed between the behavioral SAGT indices (Self-Repi, Priority-Repi, Temporal-Repi, and Efficacy-Repi) and General Decision Making Style showed a positive correlation between Temporal-Repi and a dependent decision-making style (r = 0.348, *p* = 0.047) (see Figure 4B), as well as between Efficacy-Repi and a rational decision-making style (r = 0.441, *p* = 0.010) (see Figure 4C).

On the other hand, no other significant correlations were found.

## 4. Discussion

The purpose of the current research was to adopt, for the first time, a neuroscientific perspective to explore the different cognitive and affective responses related to the self-awareness of one’s decision-making goals in a sample of professionals. To achieve this aim, a novel behavioral and ecological decision-making task (i.e., the SAGT) was designed. A neuroscientific approach was also exploited to deepen not only the explicit attributes (personality traits and decision-making style) but also the implicit neurophysiological correlates of the self-awareness of one’s decision-making goal process.

The analysis performed on behavioral, autonomic, electrophysiological, and self-report data showed the following: (i) higher self-awareness of goals in terms of time and efficacy than in terms of priority; (ii) greater activation in the temporoparietal brain area than in the frontal one for the alpha, beta, and gamma bands; and (iii) positive association between self-awareness of goals by time and dependent decision-making style (at the General Decision Making Style) and conscientiousness (measured through the 10-item Big Five Inventory), but also between self-awareness of goals by efficacy and a rational decision-making style (at the General Decision Making Style).

### 4.1. Behavioural Findings: Efficacy as the Most Diffused Implicit Key

The behavioral results revealed that every participant was able to identify and write in the first step of the SAGT at least one main decisional goal when thinking on a typical working day. The data’s variability suggests that the SAGT could effectively activate recursive thinking in an examinee and grasp individual differences in self-representation and the self-aware identification of goals relevant to the investigated construct. Examples of decisional goals identified by professionals are as follows: “meetings with collaborators on the progress of ongoing projects”, “planning the next week’s activities of the entire work team”, “creating communication content for the next event”, and “reading and responding to the email received”.

Data analysis, performed by comparing the performance of the sample in the part of the SAGT concerning the ability to self-represent their own decisional tasks and activities, also suggested that the most diffused implicit key is their efficacy, followed by the temporal sequence. These results could be analyzed by referring to the complex environment in which professionals work every day, which, together with the need to perform adequately by achieving the required outcomes, could affect the strategies adopted to prioritize one’s goals. In a dynamic context characterized by the need to cope with continuous requests and unexpected change, often with little time available, it is essential to adopt a prioritization of one’s goals in terms of time, prioritizing those referring to activities with an imminent deadline, but also always keeping in mind the efficacy of the task itself [18].

### 4.2. EEG and Autonomic Findings: Self-Representation of Decision-Making Goals as a Cognitive Process

The second finding of this study concerned the significant presence of alpha, beta, and gamma bands in the temporoparietal area of the brain during the SAGT execution. First, it is essential to point out how the presence of significant activity of these frequency bands led to the consideration that a primarily cognitive, rather than emotional, response was involved in the processes of the self-representation of decision-making goals. In agreement with the literature, the EEG delta and theta bands were previously associated with the stimulus or information emotional processing, also in the decision-making domain [45,46]. For instance, Balconi and colleagues have shown that higher values in the theta band are associated with emotionally charged states due to the decision-making process in a social setting and a sense of belonging [46]. On the other hand, the alpha, beta, and gamma bands can usually be associated with cognitive information processing. Since the task requires a memory recall of one’s decision-making goals, the significant lower presence of alpha power in the frontal regions could be interpreted as an index of the state of internally oriented information processing marked by mental simulation rather than bottom-up processing driven by the stimulus itself [42]. The beta and gamma bands’ activation could also be interpreted as a cognitive index of the activation of the neural networks involved in memory maintenance and decision making [43,44]. Indeed, the task required recalling, maintaining in memory, and selecting a rule for ordering the different decisional goals. In line with this, Kaiser and colleagues demonstrated how a short-term spatial memory paradigm activates oscillatory activity in the gamma band as a correlate of cognitive processes [44].

Another important finding in EEG data concerns the localization of the augmented beta and gamma power, namely the bilateral temporoparietal brain regions, which, according to previous studies, are involved in the self-representation mechanism, but also in the cognitive elaboration processes due to stimulus–context integration [47,48,49]. Indeed, for example, Jackobs and colleagues found a steady increase in brain activity predominantly in the temporoparietal junction in the presence of higher demands for stimulus–context integration [48].

Furthermore, the lack of results for the autonomic indices can be interpreted in a twofold way. A first potential explanation relates to the fact that professionals may have developed adequate abilities to manage and tolerate the stress response linked to any unexpected request (such as for instance, the request to list their daily goals in a restricted amount of time). In fact, since job stress has been associated by Jarczok and colleagues with autonomic nervous system imbalances [50], the absence of significant differences in autonomic measures might indicate that there are not high levels of stress. The second interpretation supports the results observed in the EEG data. Since autonomic indices provide insight into emotional processes such as arousal, stress, and anxiety [31,32,33], the lack of significant variations in these indices could suggest that the self-representation of one’s decision-making goals is mainly a cognitive process that may marginally involve emotional processing.

### 4.3. Correlation Findings: Professionals Are Success-Oriented People

Finally, the correlation between behavioral indices and self-report measures revealed that this novel task, designed to investigate the awareness of decision-making goals, can be considered a useful way to support the behavioral study of decision-making styles. Specifically, we found a positive relation between the conscientiousness personality trait measured in the 10-item Big Five Inventory and the tendency of individuals to self-represent their own goals by adopting temporal sequence criteria. This result could suggest a higher tendency of preferring time criteria in individuals that adopt planned behavior and have good organizational skills, with a system-oriented view of an organization [21,51]. In addition, we observed a relationship between a rational decision-making style and the tendency of individuals to self-represent their own goals by adopting the criteria of efficacy. This relationship could suggest that professionals who organize their goals according to the implicit key of efficacy are success-oriented people who carefully evaluate all the alternatives for making a decision and are able to consider the different possible developments of the choice taken [20,51]. This evidence paves the way for an in-depth study of personality traits, decision-making styles, and the tendency to self-represent one’s goals according to a specific implicit criterion, which translates into distinct evidence at the behavioral and neurophysiological levels.

## 5. Conclusions

To conclude, to the best of our knowledge, this research explored professionals’ cognitive and affective responses connected to the self-awareness of one’s decision-making goals by exploiting, for the first time, a combined behavioral and neurophysiological approach. The results showed that professionals were able to self-represent their goals, and the prevalent implicit key used to self-represent them is their efficacy and time. Additionally, this study highlighted how this novel and ecological decision-making task (the SAGT), which requires participants to self-represent their decision-making targets and sort them according to distinct criteria (priority, time, and efficacy), entails cognitive processing supported by frequency bands and the activation of specific neural areas that deal with memory, attention, and the synthesis of multiple types of information. Finally, this study highlights the value of integrating behavioral results with neurophysiological and autonomic evidence as a best practice for capturing valuable information on the cognitive load and emotional engagement during different phases of the decision-making process in professional contexts, including the first step of having self-awareness of one’s decision aims. This information could reflect the strengths and weaknesses of a decision maker and can be useful for identifying target aspects for tailor-made neurocognitive enhancement protocols, to even be applied in professional contexts.

Despite the novelty of this research, which attempts to fill an existing gap in the literature on the study of decision making, some limitations should be considered. The first limitation regards the selected sample: to improve the representativeness and reliability of the results, future studies should recruit samples from different and multiple organizations (such as economists, computer scientists, or entrepreneurs) to highlight possible differences in the ability and method of self-representation of decisional goals due to different work cultures and contexts. Similarly, it would also be interesting to operate a comparison and explore decision-making self-representation ability in a non-professional sample, such as university students. This would also help to disentangle the interpretation of the lack of significant differences in the autonomic indices. In fact, it might be possible that, compared to professionals, students or other population categories would display a different pattern of cognitive and emotional responses during the self-representation of their own decisional goals. On the other hand, the absence of autonomic variations in the group of students could also confirm the prevalent cognitive rather than emotional response to this task. Future research could also consider controlling or exploring the contribution of gender variables to the self-representation of one’s decisional goals process. Future studies could use multichannel electrophysiological instruments to collect more comprehensive data on brain activity, although this choice could have an impact on the ecological validity of the study itself.

Finally, it might be desirable to develop other studies that use a quali-quantitative approach and consider other investigative tools, to explore how aspects such as the emotional component, the impact of cultural factors, or prior experience may impact decision-making processes. For example, semistructured interviews or other self-report measures, such as the Maximization Scale, aimed at investigating individual differences in maximizing one’s choice, could be used to assess a subject’s subjective experience [52]. Regarding the neuroscientific approach, research protocols using multiple neurophysiological techniques, such as EEG integrated with functional near-infrared spectroscopy (fNIRS), could be implemented to better understand the implicit component of decision making.

## Data Availability

The data presented in this study are available on request from the corresponding author.
